# Causal inference study of PRRSV-MLV vaccine dosing effects on wean-to-finish performance during outbreaks

**DOI:** 10.3389/fvets.2025.1575029

**Published:** 2025-07-24

**Authors:** Swaminathan Jayaraman, Tyler Bauman, Amy Maschhoff, Caleb Shull, Peng Li, Edison Magalhaes, Giovani Trevisan, Daniel C. L. Linhares, Chunlin Li, Gustavo S. Silva

**Affiliations:** ^1^Department of Veterinary Diagnostic and Production Animal Medicine, College of Veterinary Medicine, Iowa State University, Ames, IA, United States; ^2^The Maschhoffs Ltd., Carlyle, IL, United States; ^3^Department of Animal Science, College of Agriculture and Life Sciences, Iowa State University, Ames, IA, United States; ^4^Department of Statistics, College of Liberal Arts and Sciences, Iowa State University, Ames, IA, United States

**Keywords:** porcine reproductive and respiratory syndrome virus, modified live virus vaccines, causal inference, TMLE, swine production, vaccine efficacy

## Abstract

Porcine Reproductive and Respiratory Syndrome Virus (PRRSV) greatly impacts swine production, and vaccination is the main method for reducing its economic effects on grow-finish populations. To cut costs, some producers use half-doses of modified live virus (MLV) vaccines, but the effectiveness of this approach during disease outbreaks is not well understood. This retrospective observational study used causal inference techniques to assess the impact of full-dose versus half-dose PRRSV-MLV vaccination on mortality and other key production outcomes in growing pigs experiencing PRRSV-2 outbreaks. Data analysis included 158 pig groups (47 nurseries, 111 finishing) from the Midwest United States that experienced PCR-confirmed PRRSV-2 outbreaks between 2021 and 2022, predominantly with L1C and L1A lineages. Mortality was established as the primary outcome, with cull rates, average daily gain, veterinary medicine costs, and percentage of grade A pigs at market as secondary outcomes. Using targeted maximum likelihood estimation (TMLE), a doubly robust causal inference technique, the study estimated the causal effects of vaccination dosage while accounting for potential confounders, including season, year, vaccine type, timing of vaccination, nursery stocking density, and presence of concurrent diseases. The analysis revealed distinct phase-specific effects: in the nursery, full-dose vaccination was associated with higher mortality difference (8.84, 95% CI: 4.7, 12.98) and increased veterinary costs (1.52 dollars/pig, 95% CI: 1.13, 1.91). However, in the finishing phase, full-dose vaccination significantly reduced the mortality difference (−3.40, 95% CI: −4.66, −2.29) despite slightly higher veterinary costs (0.47 dollars/pig, 95% CI: 0.03, 0.9). No significant differences between dosing strategies were observed in average daily gain, cull rates, or percentage of grade A pigs at the market. These findings suggest that while nursery groups vaccinated with full-dose had higher mortality and costs, it provided protective benefits during the economically critical finishing phase. For swine producers and veterinarians, these results indicate that the economic advantage of half-dose vaccination strategies should be carefully weighed against the increased mortality, particularly in systems with recurring PRRSV challenges. This study demonstrates the value of causal inference methods in analyzing real-world vaccination outcomes and provides evidence-based guidance for optimizing PRRSV vaccination protocols in commercial swine production.

## 1 Introduction

The swine industry continues to grapple with the persistent threat of the porcine reproductive and respiratory syndrome virus (PRRSV). This viral pathogen significantly impacts animal health and production efficiency worldwide (1–3) and is divided into two genotypes: commonly known as PRRSV type 1 (PRRSV-1), mainly comprised of viruses from Europe, and PRRSV type 2 (PRRSV-2), mainly comprised of viruses from North America (4). According to the new virus taxonomy, PRRSV includes two species: Betaarterivirus suid 1 (with virus name PRRSV-1, previously known as the European genotype) and Betaarterivirus suid 2 (with virus name PRRSV-2, previously known as the North American genotype) (5). Recent years have witnessed the emergence of highly virulent PRRSV variants, such as the 1-4-4 L1C.5 strain, which has demonstrated increased mortality, more severe clinical signs, and potentially higher transmissibility compared to other circulating strains (6). Vaccination remains a cornerstone of PRRSV control strategies in endemic populations, with production systems and veterinarians experimenting and testing different vaccination protocols under field conditions, given the ongoing challenges (7).

PRRSV vaccination strategies encompass multiple factors, including dose, timing of vaccination, which can vary from processing to several weeks post-processing depending on management practices, and vaccine type, which must be balanced against practical production realities and economic considerations (8). In the United States, commercial modified live virus (MLV) vaccines are labeled for intramuscular administration, with each brand’s dose defined by safety and efficacy studies (9, 10). However, due to various reasons like cost reduction, current marketing prices, field observations, and perception of vaccine potency led producers to explore different vaccination protocols, such as half-dose strategies.

The effectiveness of half-dose versus full-dose vaccination protocols, particularly during outbreaks, requires careful evaluation under field conditions where pigs face multiple health challenges. However, peer-reviewed evidence comparing modified dosing protocols under actual field outbreak conditions remains limited, particularly studies using causal inference methods that can account for confounding in observational data. Growing pigs may face viral challenges before or after vaccination, potentially overwhelming vaccine-induced immunity (11). The immune response to PRRSV vaccination is complex and can be influenced by various factors including the timing of vaccination, viral strain, and host factors (8). The occurrence of post-vaccination PRRSV outbreaks can significantly impact vaccine performance, as distinct viral strains may challenge the effectiveness of protective immunity conferred by vaccination (12).

While randomized controlled trials represent the gold standard for assessing interventions, they are not always feasible in commercial swine production settings due to the high costs and logistical complexities of running controlled clinical trials. Causal inference methods are advanced analytical approaches designed to estimate causal effects from observational data by systematically addressing confounding and selection bias. Unlike traditional statistical methods that focus on associations, causal inference techniques specifically aim to determine whether an observed relationship represents a true causal effect. Common causal inference methods include propensity score matching, inverse probability weighting, instrumental variables, regression discontinuity, and targeted maximum likelihood estimation (TMLE). These methods are particularly valuable when randomized controlled trials are not feasible, as they can help approximate causal effects by carefully controlling for measured confounders. The swine industry’s comprehensive data collection practices, combined with routine diagnostic information, provide a rich source of observational data. When analyzed using causal inference methods, these data can offer valuable insights into health intervention effectiveness under field conditions (13).

Despite the widespread use of PRRSV MLV vaccines in swine production, there remains limited peer-reviewed evidence evaluating the impact of modified vaccination protocols under field conditions, particularly during outbreaks. Field conditions present numerous confounding variables that cannot be controlled, such as management practices, environmental factors, concurrent diseases, and genetic variations, which can create misleading associations when using traditional analytical approaches. Most existing research relies on traditional statistical methods that may not fully account for the complex confounding present in real-world settings. This study aimed to assess differences in mortality and other key production indicators, such as cull rate, grade A percentage, veterinary medicine costs, and average daily gain, between groups vaccinated with a full-dose or half-dose of a PRRSV-modified live virus vaccine in growing pigs that experienced PRRSV outbreaks using retrospective observational data.

## 2 Materials and methods

### 2.1 Study design

This retrospective observational study assessed the impact of PRRSV-MLV vaccine doses (full vs. half) on mortality and other production outcomes in growing pigs that experienced clinical PRRSV outbreaks. The study included data from a commercial multi-site production system following sow farm to nursery to finisher progression with nursery and finisher farms located in the states of Missouri, Iowa, and Illinois between 2021 and 2022. All participating farms operated under an all-in-all-out (AIAO) management system at the farm level, with complete sanitation between batches. However, lot management between nursery and finisher phases involved complex pig movements including lot splitting, combining, and transfers between facilities. Individual nursery lots could contain pigs from multiple source sow farm flows, and at the nursery-to-finisher transition, some lots remained intact while others were redistributed through splitting or combining strategies. This complex lot management system was accounted for in our analysis through the inclusion of production flows and splitting into production phases, enabling control of genetic and health background variations. The exposure variable was the PRRSV vaccination dosage, defined as either half dose or full dose. Mortality was established as the primary outcome, with secondary outcomes including cull percentage, average daily gain, veterinary medicine costs, and percentage of grade A pigs at market (Table 1).

**Table 1:** Key variables used in the study and the notations with the descriptions.

Variable notation	Variable type	Variable name	Variable description
Y	Outcomes	Mortality percentage (primary outcome)	The percentage of pigs that died in each lot divided by the number of pigs placed.
		Cull percentage (secondary outcome)	The percentage of pigs removed from the herd due to poor health or performance in each lot during the finishing phase, divided by the number of pigs placed in each lot during the finishing phase.
		Grade A percentage (secondary outcome)	The percentage of pigs that met the highest quality grade post marketing in each lot at the end of the finishing phase
		Vet medicine costs (secondary outcome)	The total veterinary and medicine costs per pig in each lot.
		Average daily gain (secondary outcome)	The average daily weight gain of pigs in kilograms in each lot.
A	Exposure	Vaccine dose	The dose of PRRSV vaccine administered (full or half dose)
		Half-dose (0)	Received half-dose vaccine
		Full dose (1)	Received full-dose vaccine
L	Confounders	Season	The season when pigs were first placed at the site where the PRRS break occurred
		Year	The year when pigs were first placed at the site where the PRRS break occurred
		Vaccine type	The specific PRRSV vaccine product used (e.g., Prevacent, Ingelvac, Fostera)
		Timing of vaccination	When the vaccine was administered (e.g., during processing, 1-week post-processing, 2-week post-processing)
		Nursery stocking density	Number of animals per unit area (animals/ft.^2^). Calculated as number of animals in a pen divided by the total pen area.
		PRRS strain	The specific strain of PRRSV identified (based on ORF5 lineage)
		Other diseases	Presence of other diseases like PEDV, Ileitis, PDCoV, PCV2 in the lot, confirmed by PCR detection and veterinary evaluation.
		PED	1 – Yes 0 – No
		Ileitis	1 – Yes 0 – No
		PDCoV	1 – Yes 0 – No
		PCV2	1 – Yes 0 – No
U	Random intercept	Flow	The production flow associated with pigs from sow farm to wean-to-finish sites

**Figure 1:**
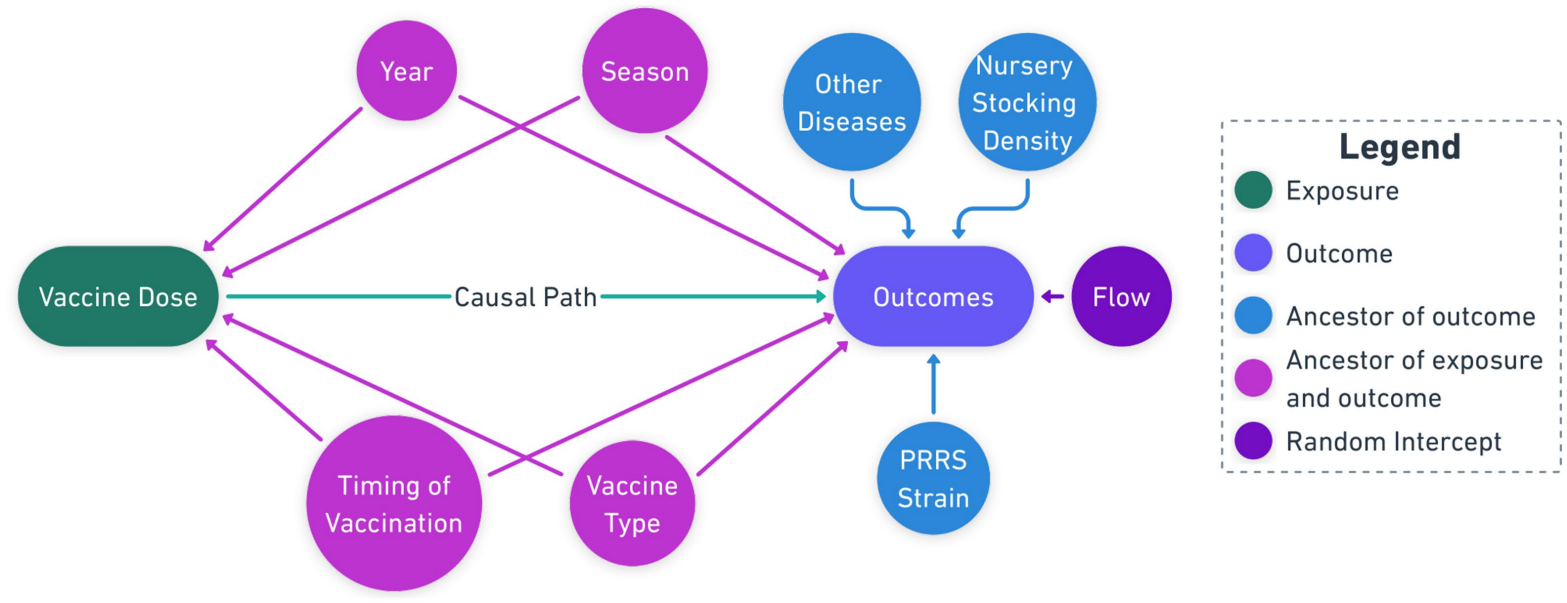
Causal Diagram showing the causal pathways between vaccine dose and confounding factors interfering with production outcomes, e.g., mortality and culls.

Eligible study groups had a reported clinical PRRSV outbreak post-placement, defined as groups exhibiting clinical signs consistent with PRRSV infection (respiratory symptoms, lethargy, decreased appetite, and/or increased mortality) as determined by attending veterinarians, followed by laboratory confirmation through PRRSV reverse-transcriptase polymerase chain reaction (RT-PCR) diagnostic testing. All diagnostic testing for this study was conducted exclusively at the Iowa State University Veterinary Diagnostic Laboratory (ISU VDL) following the production system’s standardized diagnostic protocol. The ISU VDL uses a cycle threshold (ct) value of less than 37 cycles as the cutoff for determining PRRSV-positive samples by real-time PCR. Additionally, PRRSV open reading frame-5 (ORF5) sequencing was performed at ISU VDL to determine viral lineage and restriction fragment length polymorphism (RFLP) patterns for strain identification. To uncover the causal structure from the observational data, academic and field experts were consulted to identify the relationship between variables. Using this expert knowledge, a directed acyclic graph (DAG) was constructed following established epidemiological principles for causal diagram (14) to visualize potential confounding pathways and guide the analysis (Figure 1). The study also incorporated detailed observations from field veterinarians and swine production managers, supplementing the quantitative data and providing a holistic view of PRRSV vaccination strategies.

All PRRS outbreaks occurred post-placement, with no groups experiencing outbreaks before or at the time of initial placement. Due to the complex lot redistribution system between production phases, nursery and finisher phases were analyzed as separate analytical units for the causal inference analysis.

The causal diagram visually represents the hypothesized relationships between the vaccination dosage, potential confounding factors, and the outcomes (Figure 1). The central exposure variable (green node) represents the vaccination dosage administered according to manufacturer label recommendations: full dose (Prevacent: 1 ml; Ingelvac and Fostera: 2 ml) versus half dose (Prevacent: 0.5 ml; Ingelvac and Fostera: 1 ml). The outcomes of interest (violet node) include the primary outcome of mortality percentage and secondary outcomes of cull percentage, average daily gain, medicine costs, and grade A percentage. The causal path (green arrow) represents the hypothesized direct effect of the vaccination dosage on the outcomes.

Potential confounders (purple nodes) include season and year when pigs were first placed at the outbreak site, vaccination timing (during processing, 1-week post-processing, or 2-week post-processing), and vaccine product (Prevacent PRRS MLV - Elanco, Ingelvac PRRS MLV - Boehringer Ingelheim, Fostera PRRS MLV - Zoetis). Effect modifiers (blue nodes) include the PRRSV strain (ORF5 Lineage), nursery stocking density and concurrent infections with *Lawsonia intracellularis*, porcine epidemic diarrhea virus (PEDV), porcine deltacoronavirus (PDCoV), and porcine circovirus type 2 (PCV2). The production flow from sow farm to wean-to-finish sites (amethyst node) was included as a random effect.

The outcomes cull rate and grade A percentage only apply to the finisher phase as part of the production closeouts. The cull percentage was not analyzed for the nursery phase because culling decisions are not standard practice during this early production stage, as the focus is on supporting piglet survival and growth. Similarly, grade A percentage was not analyzed for the nursery phase because this metric is related to the market-quality assessment of finished pigs.

Targeted maximum likelihood estimation was employed as the primary analytical method to estimate the causal effect of vaccination dosage on mortality and other outcomes while accounting for the complex relationships between variables in this observational study (15). Additional causal inference methods were also evaluated (1. Supplementary Methods) and are presented in Supplementary materials (2. Supplementary Results) to validate the robustness of our findings.

### 2.2 Data collection

The initial data collection focused on three commercial swine production systems in United States Midwestern region in the states of Missouri, Iowa, and Illinois from 2021 to 2022. The collected data encompassed production flow identification, vaccine product and administration details (vaccination timing, vaccination dosage), PRRSV ORF5 sequencing results, and pig age at disease outbreak.

### 2.3 Eligibility criteria

The source farms were characterized as PRRSV-vaccinated stable herds according to the American Association of Swine Veterinarians (AASV) classification system (16), providing a controlled backdrop for evaluating vaccination strategies. Clinical signs observed by veterinarians confirmed clinical PRRSV outbreak post-placement, and tested positive PRRSV by RT-PCR diagnostic testing and ORF5 sequencing results (Sanger technique). Production records included nursery and finishing closeout data, with precise documentation of pig placement dates providing a temporal framework for analyzing PRRSV vaccine responses. Additional health data regarding concurrent infections diagnosis with *Lawsonia intracellularis*, PEDV, PDCoV, and PCV2 were obtained from the ISU VDL database.

### 2.4 Data processing

Data processing followed a systematic three-step approach. First, data quality assessment was conducted through careful evaluation of production parameters. Given that this study specifically examined groups experiencing PRRSV outbreaks, mortality values that might appear as statistical outliers were retained in the analysis after careful evaluation, as they represent true biological outcomes during disease outbreaks rather than data anomalies. Quality control focused instead on identifying recording errors through cross-validation with source documents and verification of temporal sequences of health events. This approach ensures that the full spectrum of PRRSV impact on mortality is captured in our analysis, which is essential for understanding vaccine effectiveness during actual outbreak conditions.

The second step addressed missing data management. Observations with more than 10% missing information were excluded from the analysis. For records with partial missing data, particularly regarding PRRSV lineage, RFLP patterns, and farm characteristics, a hierarchical completion approach was employed. Initial data retrieval involved direct communication with production system personnel, followed by cross-referencing with ISU VDL diagnostic records when necessary.

The final step involved variable selection through a combined approach of expert domain knowledge and statistical analysis. Academic and field veterinarians collaborated to identify relevant variables based on biological plausibility and field experience. Selected variables were incorporated into the causal diagram (Figure 1) to identify potential confounders and establish the treatment-outcome pathway. This systematic approach to variable selection aligned with current recommendations for observational studies in veterinary epidemiology (13, 17).

### 2.5 Statistical analysis

To evaluate the protective effect of PRRSV vaccine doses (full vs. half) on PRRSV-attributed mortality (primary outcome) and other production parameters (secondary outcomes), we employed several analytical approaches such as generalized linear mixed effects model, propensity score matching, inverse probability treatment weighting and lastly TMLE as our primary method. TMLE was selected as it provides doubly robust estimation, remaining consistent if either the outcome model or propensity score model is correctly specified. This approach is particularly suited for veterinary observational studies where randomization is infeasible, as it can handle complex confounding while maintaining interpretable causal estimates. For comprehensive TMLE methodology, readers should consult van der Laan and Rubin (15) and Schuler and Rose (18).

To establish a baseline comparison, initially a linear mixed-effects model was employed to account for the hierarchical structure of our data and to estimate the effect of vaccine dose on the outcomes of interest. The model is expressed as:


$$Y_{\mathrm{ij}}=\beta_{0}+\beta_{1}A_{\mathrm{ij}}+\beta_{2}L_{\mathrm{ij}}+u_{j}+\varepsilon_{\mathrm{ij}}$$

Where: $Y_{\mathrm{ij}}$ is the outcome for observation i in flow j, $\beta_{0}\;$ is the intercept; $\beta_{1}\;$ is the fixed effect of the vaccine dose $A_{\mathrm{ij}}$ ; $\beta_{2}\;$ is a vector of fixed effects for the observed confounders $L_{\mathrm{ij}}$ ; $u_{j}$ is the random intercept for flow j; $\varepsilon_{\mathrm{ij}}$ is the error term. The observed confounders ( L ) included in the model were season, year, vaccine type, timing of vaccination, and farm status, as identified in our causal diagram. The impact of nursery stocking density, other diseases and PRRSV strains were also considered as potential confounders.

However, in observational studies, treatment groups often differ systematically on baseline characteristics, leading to confounding. To address this limitation and estimate the causal effect of vaccine dose on outcomes, TMLE was employed as the primary analytical method. TMLE is a doubly robust semiparametric method that combines machine learning for initial estimation with a targeted bias reduction step (15). This method is particularly well-suited for analyzing complex veterinary epidemiological data where multiple confounders exist, as it provides optimal bias-variance trade-off and remains consistent if either the outcome model or the propensity score model is correctly specified, making it more robust than traditional regression or propensity score methods alone (18). The implementation utilized Super Learner, an ensemble machine learning approach, for both the outcome model and treatment mechanism estimation. The Super Learner library combined simple means, generalized linear models, and generalized linear models with interactions. This ensemble approach optimizes prediction by combining these algorithms based on cross-validated performance (19).

The primary outcome (Y) was defined as mortality percentage, with secondary outcomes including cull rates, average daily gain, veterinary medicine costs, and percentage of grade A pigs at market for each lot/flow. Treatment (A) was specified as a binary vaccine dose (1 for full dose, 0 for half dose). Covariates (W) included time of vaccination, vaccine product, season, year, PRRSV lineage, nursery stocking density, and concurrent infections (PED, Ileitis, PDCoV, PCV2), as well as production flow. These covariates were selected based on our causal diagram (Figure 1) as potential confounders and important predictors.

#### 2.5.1 Initial estimation

Machine learning approach was used to estimate:

-
Q(A,W)=E[Y∣A,W]: The expected outcome given treatment and covariates, using an ensemble of simple means, generalized linear models, and generalized linear models with interactions.
-
g(A∣W)=P(A=1∣W) : The propensity score model.

#### 2.5.2 Targeting step

The initial estimates are updated using a clever covariate H(A,W) to reduce bias in the parameter of interest (ATE). This step solves the efficient influence curve equation.

The TMLE estimator for the Average Treatment Effect (ATE) is given by:


$${\mathrm{ATE}}_{\text{TMLE}}=\frac{1}{n}\sum_{i=1}^{n}[Q^{*(1,W_{i})}-Q^{*(0,W_{i})}]$$

Where $Q^{*}$ is the targeted estimate of the outcome regression after the updating step.

Group-specific means were estimated using TMLE with doubly robust adjustment for confounding. The TMLE-adjusted potential outcomes were used to calculate group-specific outcomes, with confidence intervals constructed using the variance estimate from the TMLE model (*α* = 0.05). Additional causal inference methods: Propensity Score Matching (PSM) and Inverse Probability of Treatment Weighting (IPTW) were evaluated and are presented in Supplementary materials.

All analyses were conducted using R version 4.3.0 with packages tmle (v1.5.0) and SuperLearner (v2.0-28). R code for replication is available upon request from the corresponding author. Detailed TMLE implementation followed standard procedures as outlined in van der Laan and Rubin (15). Researchers unfamiliar with TMLE should consult these foundational references and the tmle R package documentation before attempting replication.

## 3 Results

After data processing and cleaning, 25 pig groups were removed, and the final dataset consisted of 455,034 pigs placed into 158 pig groups with PRRSV-2 outbreaks (47 nurseries, 111 finishing) from 2021 to 2022. Of the 47 nursery groups, 8 (18%) received a half dose of the PRRSV vaccine, while 39 (82%) received a full dose (Table 2). These groups progressed to 111 finishing groups, where 25 (23%) received a half dose and 86 (77%) received a full dose (Table 2). All groups experienced PCR-confirmed PRRSV-2 outbreaks, with predominant lineages being L1C and L1A. No outbreaks with PRRSV-1 have been detected. The average age at PRRSV outbreak was 11 weeks post-placement, with over 90% of outbreaks occurring between 6 and 12 weeks post-placement.

**Table 2:** Frequency distribution by year and vaccine dose for nursery and finisher groups.

Year	Season	Nursery		Finisher	
		Half	Full	Half	Full
2021	Winter	0	3	2	6
2021	Spring	4	1	11	2
2021	Summer	1	0	1	0
2021	Fall	2	13	5	23
2022	Winter	0	2	0	3
2022	Spring	0	6	2	19
2022	Summer	0	3	1	10
2022	Fall	1	11	3	23

### 3.1 Causal analysis using TMLE

The analyses were performed separately for nursery and finisher groups, including data from 158 pig groups (47 nurseries, 111 finishing) across three commercial finishing regions between 2021 and 2022. Table 3 presents the estimated causal effects of full-dose versus half-dose PRRSV vaccination on mortality (primary outcome), cull rates, average daily gain, veterinary medicine costs, and percentage of grade A pigs at market (secondary outcomes).

**Table 3:** TMLE least squares means and estimated casual effects for outcomes by vaccine dose (2021–2022).

Outcome	Group	Treatment TMLE LS-Mean (95% CI)		TMLE effect (95% CI)
		Half dose	Full dose	
Mortality (%)	Nursery	8.40^a^ (4.30, 12.60)	17.30^b^ (13.10, 21.40)	8.84 (4.70, 12.98)*
	Finisher	9.90^a^ (8.70, 11.10)	6.40^b^ (5.30, 7.60)	−3.40 (−4.66, −2.29)*
Vet med costs	Nursery	2.60^a^ (2.20, 3.00)	4.10^b^ (3.70, 4.50)	1.52 (1.13, 1.91)*
	Finisher	1.00^a^ (0.80, 1.30)	1.50^b^ (1.20, 1.60)	0.47 (0.03, 0.90)*
Average daily gain	Nursery	0.50^a^ (0.40, 0.60)	0.50^a^ (0.40, 0.60)	−0.01 (−0.09, 0.08)
	Finisher	1.10^a^ (1.00, 1.10)	1.10^a^ (1.10, 1.20)	0.04 (−0.00, 0.09)
Cull (%)	Nursery	–	–	–
	Finisher	4.30^a^ (3.20, 5.50)	4.30^a^ (3.10, 5.40)	−0.05 (−1.22, 1.10)
Grade A (%)	Nursery	–	–	–
	Finisher	86.40^a^ (84.20, 88.60)	88.10^a^ (85.90, 90.20)	1.70 (−0.46, 3.86)

^a,b^Different superscript letters within rows indicate statistically significant differences between treatment groups (*p* < 0.05) based on TMLE-adjusted comparisons. *Asterisk indicates statistically significant treatment effect where the 95% confidence interval excludes zero. - Dash indicates outcome not applicable for the production phase. LS-Mean = Least Squares Mean adjusted for confounders; CI = Confidence Interval; TMLE = Targeted Maximum Likelihood Estimation.

**Figure 2:**
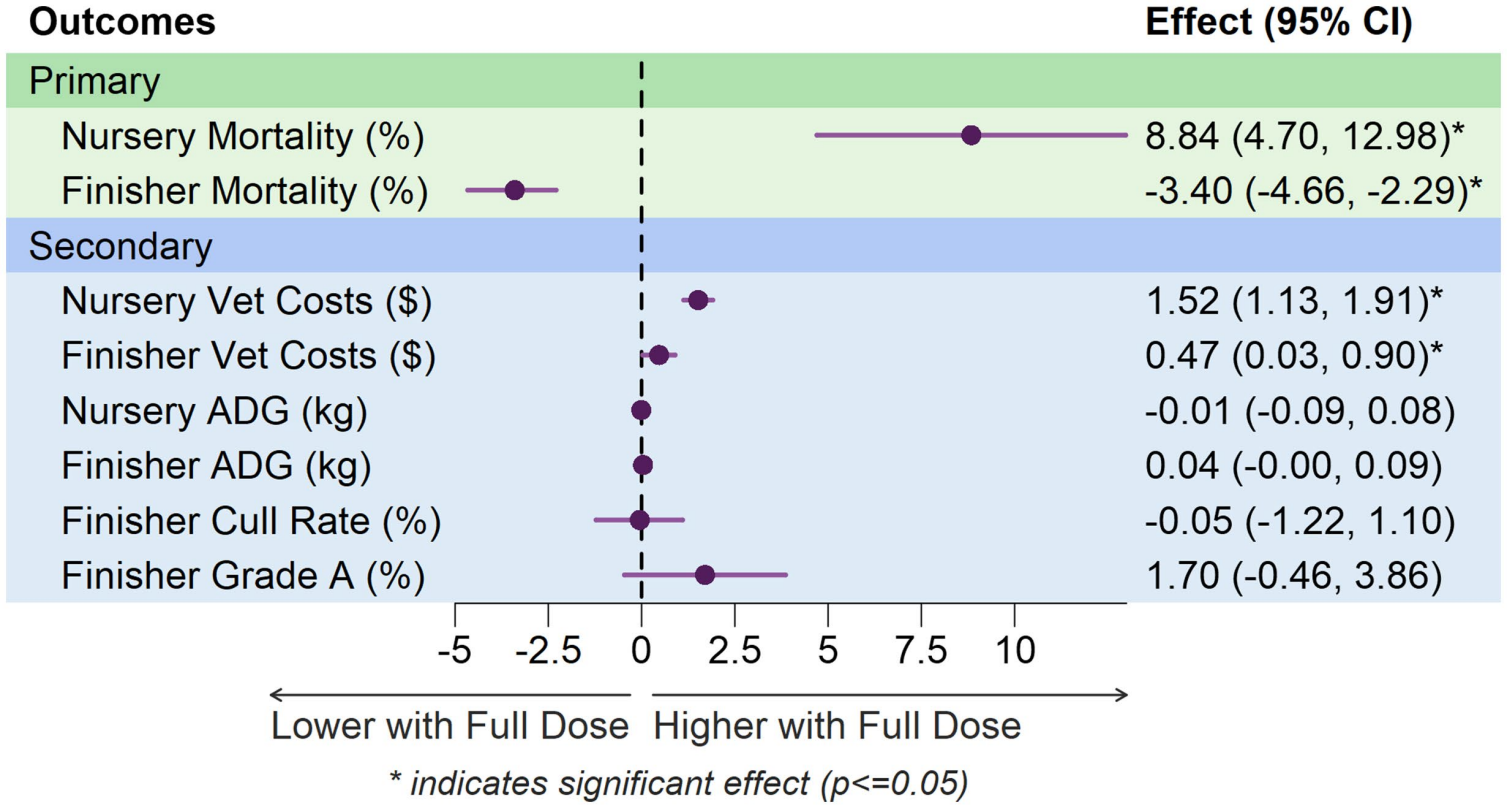
Forest plot of treatment effects comparing Full versus Half dose PRRSV vaccination.

The forest plot illustrates the phase-specific effects of vaccination dosage across all outcomes (Figure 2). Treatment effects are shown as point estimates with 95% confidence intervals. Primary outcomes (green panel) include mortality in nursery and finisher phases. Secondary outcomes (blue panel) include veterinary medicine costs, average daily gain (ADG), cull rate, and grade A percentage.

### 3.2 Treatment effects

#### 3.2.1 Mortality

In the nursery phase, TMLE analysis revealed a significantly higher mortality percentage with full doses (8.84 percentage points, 95% CI: 4.7, 12.98). However, in the finisher phase, full-dose groups had significantly lower mortality (−3.4 percentage points, 95% CI: −4.66, −2.29).

#### 3.2.2 Vet med costs

TMLE analysis showed significantly higher veterinary medicine costs in the full-dose groups in both nursery (1.52 dollars, 95% CI: 1.13, 1.91) and finisher phases (0.47 dollars, 95% CI: 0.03, 0.9).

#### 3.2.3 Average daily gain

In the nursery phase, no statistically significant difference in Average Daily Gain between full and half doses in nursery pigs from 2021 to 2022 (Table 3) was found. The point estimates are close to zero, and the confidence intervals include zero, indicating that there is no evidence of a meaningful effect of dose on ADG. However, in the finisher phase, the analysis shows a slight trend towards increased ADG with full doses, though this trend does not reach statistical significance at the conventional 0.05 level.

#### 3.2.4 Cull percentage

Culling becomes more relevant in later stages of production, particularly in the finishing phase (Table 3). In the finisher phase, the TMLE method shows no significant difference between groups that received full dose vs. those that received half dose.

#### 3.2.5 Grade A percentage

In the finisher phase, there was no statistical significance in Grade A Percentage between full and half doses in finisher pigs from 2021 to 2022 (Table 3). The point estimates suggest a slight increase in Grade A Percentage with full doses, but this difference is not statistically significant.

## 4 Discussion

This study provides compelling evidence for phase-specific effects of PRRSV-MLV vaccination dosing strategies in commercial swine production. Through the application of causal inference techniques on field data from nursery and finish groups, we identified distinct performance of full-dose versus half-dose vaccination across different production phases, particularly regarding prevention of mortality and impact of other economic outcomes.

The most striking finding was the contrasting effect of full-dose vaccination on mortality between production phases. During the finisher phase, groups receiving full-dose vaccination showed a 3.4 percentage point lower mortality rate, representing a substantial benefit during an economically critical period. The nursery phase data revealed different patterns, with full-dose vaccinated groups showing 8.84 percentage points higher mortality rates, alongside increased veterinary expenses observed in both phases. These phase-dependent outcomes suggest that the timing of immune response and its interaction with production stage-specific challenges such as stocking density may be crucial factors in vaccination effectiveness.

Although increased nursery mortality was observed in the full-dose vaccination group, this finding should be interpreted cautiously given the limited statistical power due to the small sample size in the nursery phase.

Our findings align with the field observations by the production system veterinarian that pigs vaccinated with full-dose PRRSV responded more favorably and had a more predictable mortality impact. This quantitative support for these observations using causal inference methods provide valuable insights for swine producers and veterinarians, particularly in managing the L1C.5 (1-4-4 1C PRRSV variant) and other common strains that posed significant challenges in Iowa finishers during 2021 (20). The phase-specific effects we observed support Lunney et al.’s (8) hypothesis about the complex interaction between PRRSV immunity and age-related factors. The reduced finisher mortality with full-dose vaccination particularly resonates with studies by Trus et al. (10) and Linhares et al. (9), who reported enhanced protection with standard vaccination protocols.

The use of TMLE, a doubly robust causal inference method, represents a significant advancement in analyzing field vaccination data. This approach allowed us to account for complex confounding relationships while providing interpretable effect estimates. The inclusion of multiple outcomes across different production phases offers a comprehensive view of vaccination impacts, moving beyond the traditional focus on single-phase mortality metrics.

Despite our comprehensive approach, several limitations should be noted. First, while our study included a substantial number of pig groups (158), these came from a specific geographical region and time period, potentially limiting generalizability. Second, the observational nature of the data means that unmeasured confounders may exist, although our causal inference approach helped mitigate this concern. Third, the predominance of specific PRRSV lineages (L1C and L1A) in our study period may affect the generalizability to regions with different strain prevalence.

Another important limitation of our study design was the inability to systematically track longitudinal PRRS status relationships between nursery and finishing phases due to the complex lot management system involving splitting, combining, and redistribution of pigs. Future studies would benefit from maintaining detailed nursery PRRS exposure records to assess their potential influence on finishing phase vaccination efficacy and mortality outcomes. The interaction between prior PRRS exposure and subsequent vaccination response represents an important area for further investigation.

Future studies could also explore the impact of different environmental conditions on PRRSV transmission and vaccine efficacy (11, 12). Exploring the efficacy of full-dose vaccination against emerging PRRSV strains and in different geographical contexts would further enhance our understanding of optimal vaccination strategies. For swine producers and veterinarians, these findings suggest that the economic advantages of half-dose vaccination strategies should be carefully weighed against the increased finishing mortality risk. The higher veterinary costs associated with full-dose vaccination (1.52 dollars/pig in nursery, 0.47 dollars/pig in finishing) must be evaluated against the potential benefits of reduced finishing mortality, particularly in systems with recurring PRRSV challenges. Additionally, investigating the cost-effectiveness of full-dose vaccination in different farm settings would provide valuable information for decision-making.

## 5 Conclusion

This study demonstrates the value of advanced statistical methods in analyzing real-world vaccination outcomes while highlighting the complexity of PRRSV control decisions. The phase-specific effects identified here provide evidence-based guidance for vaccination protocols in commercial swine production, though careful consideration of system-specific factors remains crucial. Future research should focus on optimizing vaccination strategies to balance early-phase impacts with late-phase benefits while considering economic implications across the entire production cycle. As the swine industry continues to face challenges from PRRSV and its variants, our results offer a data-driven approach to optimizing vaccination strategies and enhancing overall herd health management.

## Data Availability

The data that support the findings of this study are available from the corresponding author upon reasonable request. Some data may not be made publicly available due to privacy or ethical restrictions.
