# Supplementary material for: Causal inference study of PRRSV-MLV vaccine dosing effects on wean-to-finish performance during outbreaks
Source: Front Vet Sci. 2025 Jul 24;12:1575029. doi: 10.3389/fvets.2025.1575029 (PMC12330286; doi:10.3389/fvets.2025.1575029)
Supplement: Supplementary file 1 [file Supplementary_file_1.docx]

Supplementary Material

# Supplementary Methods

## Propensity Score Matching (PSM)

The propensity score, defined as the probability of receiving the full vaccine dose conditional on the observed confounders, was estimated using logistic regression:

$$\boldsymbol{e}\left( \boldsymbol{L} \right)\boldsymbol{=P}\left( \boldsymbol{A=1} | \boldsymbol{L} \right)$$

where A is the treatment indicator (1 = full dose, 0 = half dose) and L is the vector of observed confounders including Season, Year, Vaccine Type, and Timing of Vaccination, as identified in our causal diagram.

Full matching was implemented to retain all observations while creating a weighted sample where the distribution of covariates is similar between the treatment groups, following established guidelines (Randolph et al., 2019; Olmos and Govindasamy, 2019). The balance of covariates was assessed using standardized mean differences (SMD) and variance ratios.

After matching, a weighted linear mixed-effects model was fitted to estimate the treatment effect:

$$\boldsymbol{w}_{\boldsymbol{i}}\boldsymbol{Y}_{\boldsymbol{ij}}\boldsymbol{=}\boldsymbol{w}_{\boldsymbol{i}}\left( \boldsymbol{\beta}_{\boldsymbol{0}}\boldsymbol{+}\boldsymbol{\beta}_{\boldsymbol{1}}\boldsymbol{A}_{\boldsymbol{i}}\boldsymbol{+}\boldsymbol{\beta}_{\boldsymbol{2}}\boldsymbol{L}_{\boldsymbol{i}}\boldsymbol{+}\boldsymbol{u}_{\boldsymbol{j}} \right)\boldsymbol{+}\boldsymbol{w}_{\boldsymbol{i}}\boldsymbol{\varepsilon}_{\boldsymbol{ij}}$$

where $w_{i}$represents the weight for each observation $i$ derived from the propensity score matching process, $Y_{ij}$ the mortality percentage for observation $i$ in flow$j$ , $A_{i}$ is the vaccine dose, $L_{i}$ includes additional covariates such as nursery stocking density, PRRS Strain and Other Diseases, $u_{j}$ is the random intercept for flow $j$, and $\varepsilon_{ij}$ is the error term.

This approach allows us to estimate the average treatment effect (ATE) of vaccine dose on mortality while balancing observed confounders between treatment groups and accounting for the hierarchical structure of the data. However, it is essential to note that PSM can only account for observed confounders, and the validity of our causal inference relies on the assumption of no unmeasured confounding.

## Inverse Probability of Treatment Weighting (IPTW)

The IPTW approach combined propensity score weighting with outcome regression to provide unbiased estimates if either the propensity score model or the outcome model is correctly specified.

First, propensity scores were estimated using a logistic regression model:

$$\boldsymbol{logit}\left( \boldsymbol{e}\left( \boldsymbol{L} \right) \right)\boldsymbol{=}\boldsymbol{\beta}_{\boldsymbol{0}}\boldsymbol{+}\boldsymbol{\beta}_{\boldsymbol{1}}\boldsymbol{L}_{\boldsymbol{i}}$$

where $e(L)$ is the propensity score, representing the probability of receiving the full vaccine dose given the observed confounders including Season, Year, Vaccine Type, and Timing of Vaccination, as $L$.

While not typically part of standard IPTW procedures, full matching before weight calculation was incorporated to improve covariate balance further. This step creates matched sets of treated and control units, which can help reduce extreme weights and improve the stability of the subsequent IPTW analysis (Stuart, 2010). Following the matching, the inverse probability weights for each observation was calculated:

$$\boldsymbol{W=}\frac{\boldsymbol{A}}{\boldsymbol{e}\left( \boldsymbol{L} \right)}\boldsymbol{+}\frac{\boldsymbol{1-A}}{\boldsymbol{1-e}\left( \boldsymbol{L} \right)}$$

where A is the treatment indicator (1 for full dose, 0 for half dose).

Using these weights, a linear mixed-effects model was fitted to estimate the treatment effect:

$$\boldsymbol{W}_{\boldsymbol{i}}\boldsymbol{Y}_{\boldsymbol{ij}}\boldsymbol{=}\boldsymbol{W}_{\boldsymbol{i}}\left( \boldsymbol{\beta}_{\boldsymbol{0}}\boldsymbol{+}\boldsymbol{\beta}_{\boldsymbol{1}}\boldsymbol{A}_{\boldsymbol{i}}\boldsymbol{+}\boldsymbol{\beta}_{\boldsymbol{2}}\boldsymbol{L}_{\boldsymbol{i}}\boldsymbol{+}\boldsymbol{u}_{\boldsymbol{j}} \right)\boldsymbol{+}\boldsymbol{W}_{\boldsymbol{i}}\boldsymbol{\varepsilon}_{\boldsymbol{ij}}$$

where $Y_{ij}$is the mortality percentage for observation i in flow $j$, $A_{i}$ is the vaccine dose, $L_{i}$includes all other covariates (Season, Year, Vaccine Type, Timing of Vaccination, Nursery stocking density, Lineage, PED, *Lawsonia intracellularis*, Delta, PCV2), $u_{j}$ is the random intercept for flow $j$, and $\varepsilon_{ij}$ is the error term.

The implementation of this method was partially guided by the tutorial provided by McCaffrey et al. (2013). By incorporating both the propensity score model and the outcome model, this method offers protection against misspecification in either model, enhancing the validity of our causal inferences.

# Supplementary Tables

Supplementary Table 1. Least Squares Means for Outcomes by Vaccine Dose (2021-2022) (PSM and IPTW).

| **Outcome** | **Group** | **PSM LS-Mean (95% CI)** | | **IPTW LS-Mean (95% CI)** | |
| --- | --- | --- | --- | --- | --- |
|  |  | Half Dose | Full Dose | Half Dose | Full Dose |
| Mortality (%) | Nursery | 5.5 ^a^ (1.2, 26.0) | 10.8 ^a^ (3.0, 38.5) | 6.4 ^a^ (1.8, 23.0) | 14.2 ^b^ (3.3, 33.4) |
|  | Finisher | 9.9 ^a^ (4.5, 21.4) | 6.9 ^b^ (3.2, 14.8) | 10.3 ^a^ (5.9, 17.7) | 7.7 ^b^ (4.5, 13.2) |
| Vet Med Costs | Nursery | 2.0 ^a^ (0.9, 4.4) | 2.9 ^a^ (1.5, 5.7) | 2.1 ^a^ (1.1, 4.1) | 2.8 ^a^ (1.5, 5.4) |
|  | Finisher | 1.0 ^a^ (-0.6, 2.7) | 1.8 ^b^ (0.2, 3.4) | 1.0 ^a^ (-0.6, 2.7) | 1.8 ^b^ (0.2, 3.4) |
| Average Daily Gain | Nursery | 0.6 ^a^ (0.2, 0.9) | 0.6 ^a^ (0.3, 0.9) | 0.6 ^a^ (0.3, 0.9) | 0.6 ^a^ (0.3, 0.9) |
|  | Finisher | 1.1 ^a^ (0.8, 1.4) | 1.1 ^a^ (0.8, 1.3) | 0.9 ^a^ (0.7, 1.2) | 1.0 ^a^ (0.8, 1.3) |
| Cull (%) | Nursery | - | - | - | - |
|  | Finisher | 4.3 ^a^ (-3.1, 11.7) | 6.1 ^b^ (-0.8, 13.1) | 4.6 ^a^ (-0.2, 9.5) | 5.5 ^a^ (0.7, 10.3) |
| Grade A (%) | Nursery | - | - | - | - |
|  | Finisher | 82.0 ^a^ (70.9, 93.1) | 83.3 ^a^ (72.9, 93.7) | 81.0 ^a^ (72.0, 90.0) | 81.8 ^a^ (72.8, 90.7) |

Supplementary Table 2. Estimated Causal Effects of Full-Dose vs. Half-Dose PRRSV Vaccination (2021-2022) (PSM and IPTW).

| **Outcome** | **Group** | **PSM Effect (95% CI)** | **IPTW Effect (95% CI)** |
| --- | --- | --- | --- |
| Mortality (%) | Nursery | 5.3 (1.9, 12.5) | 7.8 (1.5, 10.4)* |
|  | Finisher | -3.0 (-1.3, -6.6)* | -2.6 (-1.5, -4.5)* |
| Vet Med Costs | Nursery | 0.9 (0.5, 1.33) | 0.7 (0.4, 1.2) |
|  | Finisher | 0.8 (0.9, 0.7)* | 0.8 (0.9, 0.7)* |
| Average Daily Gain | Nursery | 0.0 (0.0, 0.0) | 0.0 (0.0, -0.0) |
|  | Finisher | -0.0 (0.0, -0.0) | 0.1 (0.1, -0.9) |
| Cull (%) | Nursery | - | - |
|  | Finisher | 1.8 (2.3, 1.4)* | 0.9 (0.8, 0.9) |
| Grade A (%) | Nursery | - | - |
|  | Finisher | 1.3 (0.6, 2.0) | 0.7 (0.7, 0.8) |

# Supplementary References

1. Ji, J., Wang, C., He, Z., Hay, K. E., Barnes, T. S., and O’Connor, A. M. (2020). Comparing the estimates of effect obtained from statistical causal inference methods: An example using bovine respiratory disease in feedlot cattle. *PLoS One* 15, e0233960. doi: 10.1371/journal.pone.0233960
2. McCaffrey, D. F., Griffin, B. A., Almirall, D., Slaughter, M. E., Ramchand, R., and Burgette, L. F. (2013). A tutorial on propensity score estimation for multiple treatments using generalized boosted models. *Stat. Med.* 32, 3388–3414. doi: 10.1002/sim.5753
3. Olmos, A., and Govindasamy, P. (2019). A Practical Guide for Using Propensity Score Weighting in R. *Practical Assessment, Research, and Evaluation* 20, 13. doi: 10.7275/jjtm-r398
4. Randolph, J. J., Falbe, K., Manuel, A. K., and Balloun, J. L. (2019). A Step-by-Step Guide to Propensity Score Matching in R. *Practical Assessment, Research, and Evaluation* 19, 18. doi: 10.7275/n3pv-tx27
5. Stuart, E. A. (2010). Matching methods for causal inference: A review and a look forward. *Stat. Sci.* 25, 1–21. doi: 10.1214/09-STS313
